# Surgical treatment and perioperative management of intracranial aneurysms in Chinese patients with ischemic cerebrovascular diseases: a case series

**DOI:** 10.1186/s12883-018-1147-8

**Published:** 2018-09-14

**Authors:** Yangrui Zheng, Chen Wu

**Affiliations:** Department of Neurosurgery, Hainan Branch of Chinese People’s Liberation Army General Hospital, Sanya, China

**Keywords:** Intracranial aneurysms, Ischemic cerebrovascular diseases, Perioperative management, Surgical treatment

## Abstract

**Background:**

Patients with ischemic cerebrovascular diseases are more likely to suffer from intracranial aneurysms, and their surgical treatment has a growing controversy in this condition. The current case series was aimed at exploring surgical treatment and perioperative management of intracranial aneurysms in Chinese patients with ischemic cerebrovascular diseases.

**Methods:**

Minimally invasive surgical approach through small pterion or inferolateral forehead was applied in 31 patients. Anti-platelet drugs were withdrawn 1 week before surgical operation. Systolic blood pressure was controlled to be more than 110 mmHg and increased by 20% after the clipping of intracranial aneurysms. Branches of external carotid artery were spared to ensure collateral circulation. Temporary blocking was minimized and ischemic time was shortened during surgical operation.

**Results:**

Patients had an average age of 66 (46–78) years, and proportion of males was 39% (12 males). There were 35 unruptured intracranial aneurysms with a diameter more than 5 mm. There were 20 posterior communicating and anterior choroidal aneurysms (57%), seveb middle cerebral aneurysms (20%), and eight anterior communicating aneurysms (23%), with 21 lobular aneurysms (60%). Twenty-nine patients had normal neurological function (Glasgow Outcome Scale [GOS] 5), one patient with mild neurological defect (GOS 4), and one patient with severe neurological defect (GOS 3) at discharge. Meanwhile, there were 26 patients with modified Rankin Scale (MRS) 0–1, 4 patient with MRS 2, and one patient with MRS 3 at discharge. There were four patients lost during the follow-up. During the follow-up, 26 patients had normal neurological function (GOS 5), and one patient with severe neurological defect (GOS 3). Meanwhile, there were 25 patients with MRS 0–1, one patient with MRS 2, and one patient with MRS 3. All patients had no recurrence of intracranial aneurysms after operation.

**Conclusions:**

The current case series found that minimally invasive surgical approach and intraoperative monitoring, supplemented by effective management of cerebrovascular perfusion, circulation and coagulation, can promote the treatment of intracranial aneurysms and prevent the development of cerebral ischemia and aneurysm rupture in Chinese patients with ischemic cerebrovascular diseases. Future studies with large sample size will be needed to confirm the results from the current case series.

## Background

Ischemic cerebrovascular diseases lead to transient ischemic attack (TIA) and irreversible cerebral infarction, and cerebral infarction has a mortality rate of 30–40% [1]. Meanwhile, intracranial aneurysms are one kind of severe intracranial disease mainly responsible for subarachnoid hemorrhage, and the rupture of intracranial aneurysms results in a similar mortality rate of 30–40% [2–4]. Concurrence rate of intracranial aneurysms and ischemic cerebrovascular diseases was 0.5–5% [5, 6]. Patients with ischemic cerebrovascular diseases are more likely to suffer from intracranial aneurysms because of the change in arterial structure and function [7].

Intracranial aneurysms and ischemic cerebrovascular diseases have different disease characteristics and treatment methods, and therefore, when patients with ischemic cerebrovascular diseases had intracranial aneurysms, their surgical treatment is at the center of a growing controversy [8]. If the treatment of intracranial aneurysms precedes that of ischemic cerebrovascular diseases, perioperative hypoperfusion can induce the ischemic events and even fatal cerebral infarction with a large area [9]. If the treatment of ischemic cerebrovascular diseases precedes that of intracranial aneurysms, rapid increase in intracranial blood flow can induce the rupture of intracranial aneurysms [10]. The current case series was aimed at exploring surgical treatment and perioperative management of intracranial aneurysms in Chinese patients with ischemic cerebrovascular diseases.

## Methods

### Patients

In the current case series, all patients with not only intracranial aneurysms with a diameter more than 5 mm, but also cervical or intracranial arterial stenosis or occlusion (> 50%), were diagnosed on the basis of computed tomography angiography (CTA) or digital substraction angiography (DSA), and received surgical treatment of intracranial aneurysms in our hospital between April 2010 and April 2014. The existence of symptoms and signs, such as dizziness, headache and nerve localization signs, also supported clinical diagnosis. Patients with recurrent TIA, soft atherosclerotic plaques or ulcer formation were excluded from the current case series. Therefore, there were 31 patients in the current case series.

### Surgical treatment

In the current case series, patients were treated based on clinical practice but not as part of a prospective, controlled study. Minimally invasive surgical approach through small pterion or inferolateral forehead was applied in all patients. Intracranial aneurysms were treated with clipping. Surgical operation was monitored by electroencephalogram and somatosensory evoked potential.

During surgical operation, blood flow and its patency were evaluated by microvascular Doppler ultrasound. Fluorescence angiography was applied to evaluate the patency of parental artery and perforating artery. Contralateral aneurysms and ipsilateral aneurysms were defined according to the locations of intracranial aneurysms and arterial stenosis or occlusion. For communicating aneurysms, contralateral aneurysms and ipsilateral aneurysms were defined based on the advantage of blood supply.

### Perioperative management

Anti-platelet drugs were applied until 1 week before surgical operation and resumed on the second day after surgical operation. In order to satisfy cerebral blood perfusion of patients with ischemic cerebrovascular diseases, systolic blood pressure was controlled to be more than 110 mmHg and increased by 20% after the clipping of intracranial aneurysms. Postoperative blood pressure was controlled to be not lower than daily level. Branches of external carotid artery, such as superficial temporal artery and middle meningeal artery, were spared to ensure collateral circulation. Temporary blocking was minimized and ischemic time was shortened during the operation.

In order to avoid the rupture of atherosclerotic plaques, these plaques should be kept away from and surgical operation should be light and soft to the largest extent when clipping intracranial aneurysms. Papaverine soaking was locally applied after the clipping of intracranial aneurysms. Low molecular weight dextran and nimodipine were applied to improve the microcirculation. Before surgical operation and during the follow-up, CTA or DSA was applied to observe arterial stenosis and intracranial aneurysms, and assess ischemic cerebrovascular capacity and tolerance. Glasgow Outcome Scale (GOS) and Modified Rankin Scale (MRS) were applied to evaluate neurological function and prognostic performance.

### Postoperative follow-up

All patients were followed up at the first month, third months and sixth months, and then once a year after surgical operation. There were 4 patients lost during the mean follow-up period of 18 (6–40) months.

## Results

Patients had an average age of 66 (46–78) years, and proportion of males was 39% (12 males). As shown in Table 1, percentages of dizziness, headache and nerve localization signs were 77, 52 and 29%, respectively. There were 28 patients with a long-term use of anti-platelet drugs. There were 35 intracranial aneurysms in 31 patients, and all intracranial aneurysms had a diameter more than 5 mm. There were 20 posterior communicating and anterior choroidal aneurysms (57%), seven middle cerebral aneurysms (20%), and eight anterior communicating aneurysms (23%). All aneurysms had no rupture, and there were 21 lobular aneurysms (60%). There were 17 contralateral aneurysms (49%) and 18 ipsilateral aneurysms (51%). Besides, there were 18 patients with carotid arterial stenosis (> 50%, nine patients with stenosis > 75%), three patients with internal carotid arterial occlusion of cervical segment, three patients with internal carotid arterial occlusion of intracranial segment, four patients with internal carotid arterial stenosis of intracranial segment (> 50%), two patients with middle cerebral arterial stenosis (> 50%), and one patients with middle cerebral arterial occlusion.

**Table 1:** Characteristics of patients, aneurysms and prognoses

Characteristics	Descriptions
Age (year)	66 (46–78)
Males, n (100%)	12 (39%)
Appearance, n (100%)	
Dizziness	24(77%)
Headache	16(52%)
Nerve localization signs	9(29%)
Number of aneurysms, n	35
Location of aneurysms, n (100%)	
Posterior communicating and anterior choroidal aneurysm	20 (57%)
Middle cerebral aneurysm	7 (20%)
Anterior communicating aneurysm	8 (23%)
Diameter of aneurysms > 5 mm, n (100%)	35 (100%)
Aneurysm rupture, n	0
Lobular aneurysm, n (100%)	21 (60%)
Contralateral aneurysm, n (100%)	17(49%)
Ipsilateral aneurysm, n (100%)	18(51%)
Carotid arterial stenosis, n (100%)	18(58%)
Internal carotid arterial occlusion of intracranial segment, n (100%)	3(10%)
Internal carotid arterial stenosis of intracranial segment, n (100%)	4(13%)
Middle cerebral arterial stenosis, n (100%)	2(7%)
Middle cerebral arterial occlusion, n (100%)	1(3%)
Glasgow Outcome Scale at discharge, n (100%)	
5	29(94%)
4	1(3%)
3	1(3%)
Modified Rankin Scale at discharge, n (100%)	
0–1	26(84%)
2	4(13%)
3	1(3%)
Glasgow Outcome Scale at follow-up, n (100%)	
5	26(84%)
3	1(3%)
Lost	4(13%)
Modified Rankin Scale at follow-up, n (100%)	
0–1	25(81%)
2	1(3%)
3	1(3%)
Lost	4(13%)

**Fig. 1:**
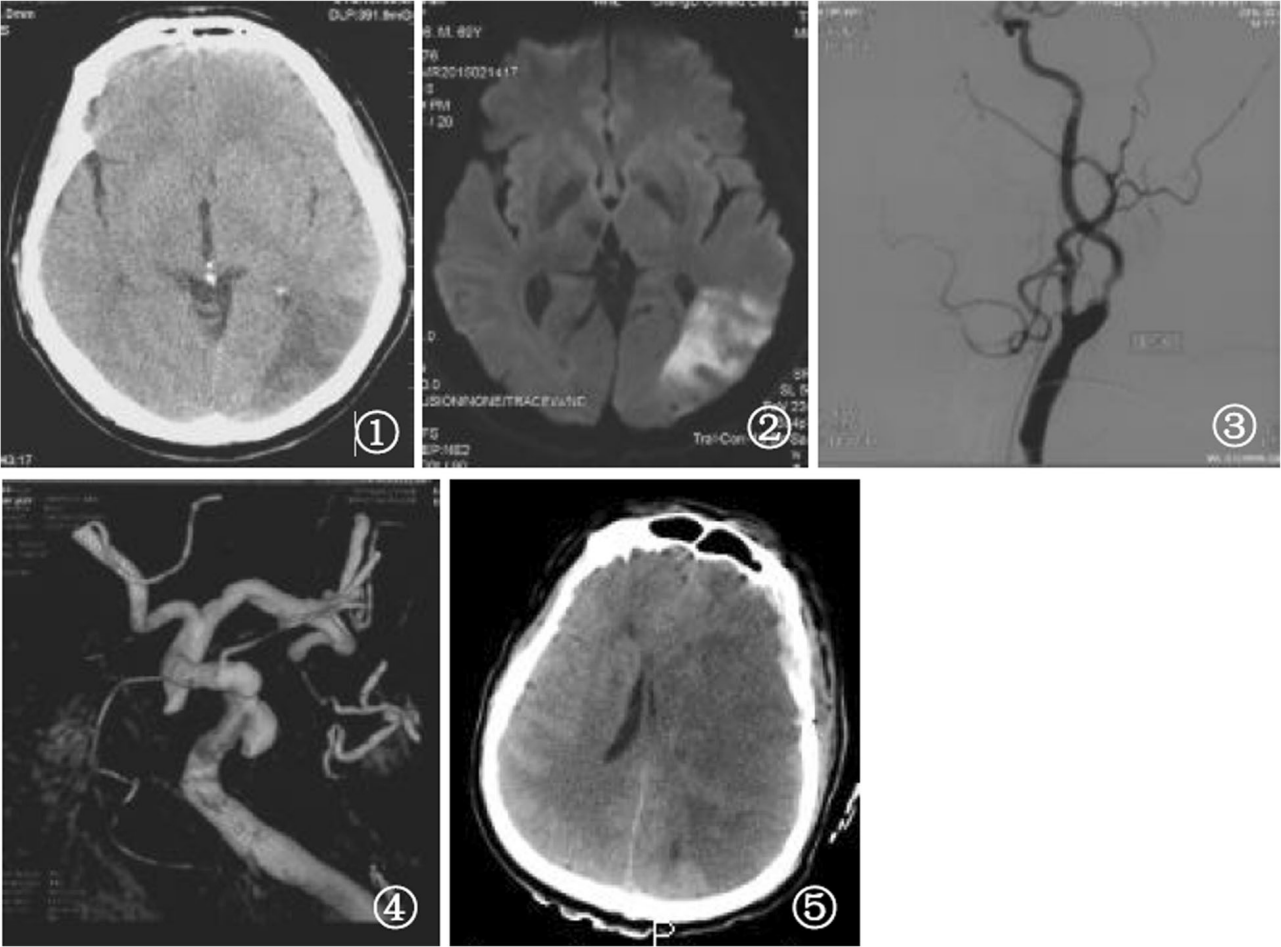
One patient, 65–70 years old. **①** cerebral infarction (left occipital lobe) shown in Computed Tomography scan before surgical operation; **②** cerebral infarction (left occipital lobe) shown in Magnetic Resonance Imaging before surgical operation; ③left carotid arterial stenosis (> 75%) shown in Digital Subtraction Angiography before surgical operation; **④** left posterior communicating aneurysms with a diameter of 5.6 mm shown in Digital Subtraction Angiography before surgical operation; **⑤** cerebral infarction (left hemisphere) with a large area shown in Computed Tomography scan after operation

One early awake patient became unconsciousness and had contralateral hemiplegia on the second day after surgical operation. Massive cerebral infarction was diagnosed according to CT scan in this patient. In spite of big bone flap excised and dura matter enlarged, hemiplegia and aphasia still existed in this patient (Fig. 1). Minor cerebral infarction in posterior limb of internal capsule occurred in another patient. Twenty-nine patients had normal neurological function (GOS 5), one patient with mild neurological defect (GOS 4), and one patient with severe neurological defect (GOS 3) at discharge. Meanwhile, there were 26 patients with MRS 0–1, four patients with MRS 2, and one patients with MRS 3 at discharge. During the follow-up, 26 patients had normal neurological function (GOS 5), and one patient with severe neurological defect (GOS 3). Meanwhile, there were 25 patients with MRS 0–1, one patient with MRS 2, and one patients with MRS 3. There were three patients with carotid arterial stenting, and one patients with carotid endarterectomy at the third month after surgical operation. All patients had no recurrence of intracranial aneurysm.

## Discussion

Prevalence of intracranial aneurysms is obviously higher in patients with ischemic cerebrovascular diseases compared with general population [7]. Carotid arterial stenosis or occlusion on one side increases blood flow of contralateral carotid artery and cerebral arterial circle, aggravates the impact force and shear force of blood flow on the vessel wall, and results in the occurrence and development of intracranial aneurysms [11]. In South Korean patients with ischemic cerebrovascular diseases, 47.4 and 52.6% of intracranial aneurysms located on the same and other side of arterial stenosis or occlusion, respectively [7]. These results are close to our data (49 and 51%) in the current case series. Moreover, ischemic cerebrovascular diseases and intracranial aneurysms have similar etiology and pathogenesis [8]. Aging, metabolic disturbance (elevated blood pressure, glucose and lipids), and poor vessel condition accelerates the occurrence and development of intracranial aneurysms and arterial plaques [12]. Inflammatory reaction and other mechanisms should be responsible for the occurrence and development of intracranial aneurysms and arterial plaques [7].

Since it is very difficult to balance the risk of aneurysm rupture and cerebral infarction, surgical treatment of intracranial aneurysms without rupture in patients with ischemic cerebrovascular diseases has been highly controversial [8]. If the treatment of ischemic cerebrovascular diseases precedes that of intracranial aneurysms, rapid increase in intracranial blood flow can induce the rupture of intracranial aneurysms [10]. If the treatment of intracranial aneurysms precedes that of ischemic cerebrovascular diseases, perioperative hypoperfusion can induce ischemic events and even fatal cerebral infarction with a large area [9]. One meta-analysis has shown no increased risk of aneurysm rupture caused by carotid endarterectomy [13]. Other study has also suggested that carotid arterial stenting and carotid endarterectomy are not related to increased aneurysm rupture [14]. It is worth noting that 82% of intracranial aneurysms have a diameter less than 5 mm in that study [14]. Thus, ischemic cerebrovascular diseases were generally believed to receive surgical treatment before that of intracranial aneurysms with a diameter less than 5 mm.

Due to a lack of related studies, there has been debatable about sequential order of surgical treatment for ischemic cerebrovascular diseases and intracranial aneurysms with a diameter more than 5 mm. One study has found no increased prevalence of ischemic events in patients without high risk of cerebrovascular accident caused by the clipping of intracranial aneurysms with a diameter more than 5 mm [15]. Thus, surgical indications of intracranial aneurysms before ischemic cerebrovascular diseases include: 1) unruptured aneurysms with a diameter more than 5 mm; 2) without recurrent TIA, soft atherosclerotic plaques or ulcer formation; 3) aneurysm rupture and its caused subarachnoid hemorrhage.

Minimally invasive surgical approach through small pterion or inferolateral forehead is very suitable for the treatment of unruptured aneurysms in patients with ischemic cerebrovascular diseases [16]. Branches of external carotid artery, such as superficial temporal artery and middle meningeal artery, should be spared to ensure collateral circulation [9]. It is essential to minimize temporary blocking, shorten ischemic time and avoid plaque rupture. Papaverine soaking can be locally applied after the clipping of intracranial aneurysms to avoid the vasospasm.

Blood pressure is of great importance to the success of surgical operation, and should be controlled within reasonable range to avoid perioperative hypoperfusion and aneurysm rupture [4]. Preoperative communication with anesthesiologists can play a significant role in effective management of blood pressure. Postoperative blood pressure should be monitored to approach daily level to improve cerebral perfusion [2].

Patients with ischemic cerebrovascular diseases often have a long-term use of anti-platelet drugs [17]. These drugs should be withdrawn 1 week before surgical treatment, and applied on the second day after surgical treatment. Low molecular weight dextran and nimodipine can be applied to improve the microcirculation [18]. If there is an ischemic attack during the withdrawal of drugs, surgical operation should be performed with a great deal of caution.

The current case series had strength and limitation. Its strength was to explore surgical treatment and perioperative management of intracranial aneurysms in Chinese patients with ischemic cerebrovascular diseases. However, as a case series, it had a small sample size (31 patients).

## Conclusions

The current case series found that minimally invasive surgical approach and intraoperative monitoring, supplemented by effective management of cerebrovascular perfusion, circulation and coagulation, can promote the treatment of intracranial aneurysms and prevent the development of cerebral ischemia and aneurysm rupture in Chinese patients with ischemic cerebrovascular diseases. Future studies with large sample size will be needed to confirm the results from the current case series.

## Abbreviations

CTA: Computed tomography angiography
DSA: Digital substraction angiography
GOS: Glasgow outcome scale
MRS: Modified rankin scale
TIA: Transient ischemic attack
